# Spinal Concussion

**Published:** 1890-05

**Authors:** 


### Book Reviews.

**Spinal Concussion**; Surgically Considered as a Cause of Spinal Injury, and Neurologically Restricted to a Certain Symptom Group, for which is Suggested the Designation Erichsen's Disease, as one of the Traumatic Neuroses. By S. V. Clevenger, M. D., Consulting Physician to the Reese and Alexian Hospitals, Chicago, etc. Philadelphia and London: F. A. Davis. 1889. Cloth, 8vo, pp. iv., 359. Price, $2.50, net.

The author of this monograph has intended to write for the two professions of medicine and law. In its perusal one cannot fail to be impressed with the spirit of fairness which is everywhere apparent. It is as if a medical judge were reviewing the testimony of experts on a medical question, and after weighing carefully the opinions of each, he sums up and announces his own.

Few monographs have appeared, which so thoroughly reviewed the work of other men. Erichsen, Page and Oppenheim are liberally and judiciously quoted and their views fairly discussed. This is an important feature of the book, as Erichsen and Page present such widely differing opinions, that in harmony with the spirit of the book the reader must have enough presented to draw his own conclusions.

That so much of Oppenheim is included in this volume we feel adds greatly to its value, as no man of recent years has devoted more time and research to this subject than the learned Berlin neurologist.

That attorney and physician may appreciate the varied symptoms which become grouped in the disease under consideration; a large number of clinical reports by different observers in America, England and Germany are given. In this list we find genuine and simulated cases of spinal concussion. The results of litigation are also given.

Whether we accept the author's pathological view "that spastic conditions of the cord-vessels account best for spinal irritation symptoms," we must agree that he arrives logically at his conclusion.

The book is a valuable addition to the literature of the subject, is excellently printed on good paper, and yet we are compelled to express a regret that so carefully prepared a work on a scientific subject should contain such schematic plates of the spinal cord, as appear on pages 190-191. Such plates are always inaccurate and always represent things, not as they are, but as they are not, or as the artist wishes they were. Would that spinal demarcations were all in the straight lines the artist pictures; but since they are not, let us hope we may see them as they are seen in microscopic sections. J. W. P.

